# Human nevi lack distinguishing senescence traits

**DOI:** 10.18632/aging.100537

**Published:** 2013-02-26

**Authors:** Sieu Tran, Helen Rizos

**Affiliations:** Westmead Institute for Cancer Research and Melanoma Institute Australia, University of Sydney at Westmead Millennium Institute, Westmead, New South Wales 2145, Australia

Oncogene-induced senescence is a program initiated by the aberrant activation of oncogenes. Once engaged, this program irreversibly limits the proliferative capacity of cells and may potently prevent tumor formation *in vivo*. Human nevi (moles) display many features of oncogene-induced senescence; they remain growth arrested for decades, display increased p16^INK4a^ expression, stain positive for senescence-associated-ß-galactosidase (SA-ß-Gal) [1] and carry oncogenic mutations in the BRAF kinase [2].

Evidence demonstrating that human nevi may have undergone senescence is based on the accumulation of a few predictive markers (p16^INK4a^, SA-ß-gal and Ki67) that are not exclusive to the senescence program [1]. Thus, we sought to clarify whether human nevi display a consistent senescence signature by examining an expanded panel of senescence-associated markers. These markers were used to evaluate DNA damage (γ-H2AX and p53), chromatin remodelling (senescence-associated heterochromatin foci, promyelocytic leukemia protein and histone H3 lysine 9 methylation), proliferation (Ki67, p16^INK4a^), morphology (nuclear size) and SA-ß-Gal activity [3]. Importantly, to ensure the specificity of this senescence signature, the expression of these markers was evaluated in a panel of human nevi, epidermal melanocytes, primary and metastatic melanomas.

Only Ki67 distinguished nevi from melanomas. The commonly evaluated p16^INK4a^ was abundant in both nevi and primary melanomas and the most widely accepted senescence marker, SA-ß-Gal, was detected in both nevi (5/7) and metastatic melanomas (3/7) [3]. Thus, there is not sufficient evidence to define human nevi as senescent lesions.

There are also several important clinical observations that challenge the view that nevi have undergone oncogene-induced senescence. Nevus cells can be induced to proliferate *in vivo* (e.g. after partial resection or during pregnancy) and growing nevi are more likely to carry mutant BRAF ([4]; reviewed in [3]). The identification of nevi in contiguity with up to 40% of melanomas also suggests that senescence is either a poor suppressor of melanomagenesis or that a proportion of nevus cells retain proliferative potential. This is consistent with BRAF-induced benign nevi in mice. These murine nevi expressed SA-ß-Gal and p16^INK4a^, but also contained rare mitotically active cells, and were occasionally associated with melanoma tumors [5].

Our work underscores the fact that human nevi are capable of occasional, induced proliferation and transformation. This is in accordance with the strong relationship between nevus number and melanoma risk [6] and the observation that most melanomas in younger patients (<30 years) are associated with a nevus precursor [7].

Identifying more-specific senescence-associated markers may help clarify the role of senescence in arresting human naevi, but the focus on senescence has also framed current thinking and may narrow research into other pathways of arrest. Defining the tumour suppressor mechanisms that restrict nevus cell proliferation and the drivers (genetic and non-genetic) that permit escape from this proliferative arrest remains an important research priority. This is particularly relevant as rapidly changing nevi, the emergence of new nevi (with wild-type BRAF) and newly developing melanomas have been reported in melanoma patients treated with the mutant BRAF inhibitors vemurafenib and dabrafenib [8].
